# Handheld Co-Axial Bioprinting: Application to *in situ* surgical cartilage repair

**DOI:** 10.1038/s41598-017-05699-x

**Published:** 2017-07-19

**Authors:** Serena Duchi, Carmine Onofrillo, Cathal D. O’Connell, Romane Blanchard, Cheryl Augustine, Anita F. Quigley, Robert M. I. Kapsa, Peter Pivonka, Gordon Wallace, Claudia Di Bella, Peter F. M. Choong

**Affiliations:** 1 University of Melbourne, Department of Surgery, St Vincent’s Hospital Melbourne, 29 Regent Street-Clinical Science Building, 3065 Fitzroy, VIC Australia; 2 ARC Centre of Excellence for Electromaterials Science, Intelligent Polymer Research Institute, Innovation Campus, University of Wollongong, Northfields Ave, 2522 Wollongong, NSW Australia; 3 Department of Clinical Neurosciences, 5th Floor Daly Wing, St. Vincent’s Hospital, 3065 Fitzroy, VIC Australia; 4 Department of Medicine, St Vincent’s Hospital Melbourne, 3065 Fitzroy, VIC Australia; 5 Department of Orthopaedics, St Vincent’s Hospital Melbourne, 3065 Fitzroy, VIC Australia

## Abstract

Three-dimensional (3D) bioprinting is driving major innovations in the area of cartilage tissue engineering. Extrusion-based 3D bioprinting necessitates a phase change from a liquid bioink to a semi-solid crosslinked network achieved by a photo-initiated free radical polymerization reaction that is known to be cytotoxic. Therefore, the choice of the photocuring conditions has to be carefully addressed to generate a structure stiff enough to withstand the forces phisiologically applied on articular cartilage, while ensuring adequate cell survival for functional chondral repair. We recently developed a handheld 3D printer called “Biopen”. To progress towards translating this freeform biofabrication tool into clinical practice, we aimed to define the ideal bioprinting conditions that would deliver a scaffold with high cell viability and structural stiffness relevant for chondral repair. To fulfill those criteria, free radical cytotoxicity was confined by a co-axial Core/Shell separation. This system allowed the generation of Core/Shell GelMa/HAMa bioscaffolds with stiffness of 200KPa, achieved after only 10 seconds of exposure to 700 mW/cm^2^ of 365 nm UV-A, containing >90% viable stem cells that retained proliferative capacity. Overall, the Core/Shell handheld 3D bioprinting strategy enabled rapid generation of high modulus bioscaffolds with high cell viability, with potential for *in situ* surgical cartilage engineering.

## Introduction

Three-dimensional (3D) bioprinting epitomizes the fusion of biology and engineering.

The ability to design and fabricate complex structures by printing living cells and biomaterials functionalized with biological molecules is revolutionizing tissue engineering and regenerative medicine^1^, while enabling new possibilities in drug screening and toxicology^2–4^. The generation of organized 3D tissue constructs *via* a layer-by-layer deposition process that combines cells and biomaterials in an ordered and predetermined way, allows the fabrication of multi-cellular constructs where cell-cell and cell-material interactions can mimic the physiological environment and where cellular responses to stimuli are more reflective of those found *in vivo* ^5^. In recent years this technology has inspired application for *in vitro* biofabrication of cartilage tissues^6, 7^. However, challenges still exist in the development of a fully functional tissue construct that can replicate its natural counterpart^8, 9^.

An important factor in chondral tissue engineering is the choice of biomaterial for scaffolds. Early work using materials such as chitosan have given way to more tissue-compliant hydrogels based on natural polymers, such as gelatine mainly due to their cytocompatibility and constitutional relevance to mammalian tissue^10^. Furthermore, such hydrogels’ hydrophilic nature, chemical stability and biodegradability lend favourably towards their use as versatile scaffolds for 3D printing of bio-synthetic tissue constructs using appropriate cells. Addition of chemically cross-linkable side-groups such as methacrylate/methacrylamide groups to biologically-derived hydrogels such as gelatin and hyaluronic acid facilitates chemical cross linking that further expands the application scope of these materials^11^.

Crosslinking can be achieved by physical crosslinking (reversible), chemical crosslinking (irreversible) or a combination of both^12^ and promotes a robust state change of hydrogels from (viscous) liquid to semi-solid. This provides otherwise-absent structural stability in 3D hydrogel material configurations that retain native cell adhesion properties and otherwise mimic extracellular matrix. In turn, this facilitates cell encapsulation and deposition in 3D for additive biofabrication technologies such as 3D bioprinting^13–15^. In current practise, chemical crosslinking is largely accepted as the most effective, efficient and controllable method by which to generate cross-linked hydrogels with handling and mechanical stiffness properties most appropriate to their intended use^16^. The crosslinking reaction can be initiated by irradiation of a photo-initiator chemical within the hydrogel by light of a specific wavelength. This irradiation initiates a free-radical mediated polymerization reaction between the methacrylate and photo-initiator that cross-links the bio-polymer chains to form a hydrogel. The major challenge facing chemical photo-cross-linking of cell-containing hydrogels is compromised cell viability due to cytotoxic by-products generated in-process by the cross-linking chemistry^17^.

Photo-crosslinking chemistry engenders three possible sources of cytotoxicity: (i) exposure to the photo-initiator (PI) chemical itself, (ii) exposure to UV light, (iii) exposure to free radicals created through light degradation of the PI. The most deleterious effects have been shown to occur upon exposure to the PI and UV light together, suggesting that in-process evolution of free-radicals is the most damaging step of the crosslinking process^18^. Minimizing the PI concentrations and light intensity can alleviate cell toxicity but comes at the expense of longer crosslinking times (10–30 minutes), necessary to achieve adequate biomechanical properties. Prolonging the crosslinking increases time required to print cell-containing constructs, and thereby places limitations on the clinical applicability of bioprinting. Some recently reported strategies propose “pre-setting” the structure^19^, but the additional steps could make again the procedure impractical for a direct surgical application.

In our previous work we developed a novel handheld 3D printer device called “Biopen”^20^ with the aim of promoting intra-surgery *in situ* bioprinting for cartilage biofabrication. To achieve this, the bioprinting parameters of the Biopen system requires a bio-ink that: (i) sets rapidly enough to allow handheld application to the lesion by the surgeon; (ii) generates a bio-synthetic cartilage construct of sufficient stiffness to immediately withstand the forces within the intra-articular environment and (iii) delivers viable cells with the ability to form functional chondral tissue.

To overcome the challenges stated and accommodate the requirements above-mentioned for effective formation of functional synthetic cartilage, we applied a co-axial extrusion strategy to our prototyped Biopen device. Our co-axial bioprinting is designed to deliver a hydrogel of uniform chemistry (GelMa/HAMa), with mechanical stiffness sufficient to withstand compressive force at the site of chondral lesion whilst segregating cells from the PI chemistry. By its use of a single polymer chemistry, this approach expands on other current approaches that advocate use of a combination of cross-linkable materials with different base polymer chemistries to try to achieve a balance between structure and functional integrity of the printed construct. In addition, our approach links for the first time the Core/Shell principle of deposition with *in situ* clinical (at the time of surgery) application for repair of musculoskeletal tissue such as cartilage, thereby bypassing the time-consuming step of pre-surgical laboratory-based biofabrication.

In our Biopen system, the Core component of the co-axial hydrogel contains infrapatellar Adipose-derived Mesenchymal Stem/Stromal Cells (ADSCs) encapsulated within a naturally derived hydrogel (Gelatin methacryloyl/Hyaluronic acid methacrylate, GelMa/HAMa, 10%/2%)^7^. The outer Shell component of this co-axial system contains the same hydrogel (GelMa/HAMa, 10%/2%), which becomes photo-polymerizable due to the addition of the PI. Hardening of the Shell provides the structural properties that allow 3D printing, while the ADSCs are preserved in a relatively soft, cell-friendly environment inside the Core.

In this work we derive the balance of material, cells and fabrication process parameters that facilitate the process of *in situ* deposition of chondrogenic cells using our Biopen device. We evaluate in the first instance the ideal PI based on desired/ideal mechanical (stiffness) properties and *in situ* photo-rheology of photo-crosslinked GelMa/HAMa hydrogels. We then show that our co-axial Biopen deposition process enables the use of otherwise cytotoxic photo-polymerization chemistries, by which to make viable 3D cell-containing constructs with mechanical (stiffness) properties suitable for cartilage regeneration. These results demonstrate that co-axial extrusion facilitates rapid photo-cross-linking under high intensity UV-A to deposit a viable cell-containing GelMa/HAMa hydrogel and makes possible the *in situ* surgeon-mediated deposition of viable biosynthetic cartilage repair structures. Extending from this, the co-axial approach opens the scope for use of fabrication materials that are precluded on the basis of their inherent toxicity issues, from additive biofabrication of functional tissue constructs.

## Results

### Establishment of the optimal photo-initiator molecule for GelMa/HAMa bioprinting

Chondrocytes within native articular hyaline cartilage exist in a highly compressive environment. The knee joint of a human of the global average 60 kg body mass experiences some 6 to 20 MPa of force, depending on activity^21^. For this reason, the modulus of GelMa/HAMa in which the ADSCs-derived chondrogenic cells are to be delivered to the osteochondral lesion via the Biopen needs to be sufficient to withstand compressive forces within the joint so as to sufficiently protect chondrocyte development *in situ* and prevent the collapse of the implanted scaffold. Furthermore, in light of the surgical scaffold application to the lesion, the structure needs to attain maximal modulus in the shortest period of time possible. This thus becomes the first priority for choice of photo-initiator with which to induce cross-linking of the GelMa/HAMa. In conjunction with this major requirement, the methodology needs to be minimally toxic to the cells and to allow delivery of viable proliferative cells that can undergo chondrogenesis sufficiently to form functional hyaline cartilage.

#### Structure: GelMa/HAMa hydrogel stiffness

In order to obtain a GelMa/HAMa hydrogel stiff enough to use for cartilage repair upon rapid photo-crosslinking, we first focused our study on the screening of different photocuring conditions by scoring three different photo-initiators (PIs) and photocuring time.

For this purpose, we measured Young’s Modulus, by mechanical compression testing, as a function of GelMa/HAMa hydrogel exposure time to 365 nm (UV light) at high intensity (700 mW/cm^2^) using lithium-acylphosphinate (LAP), IRGACURE2959 (IRGA) and VA086 at the same concentration (0.1% w/v).

**Figure 1:**
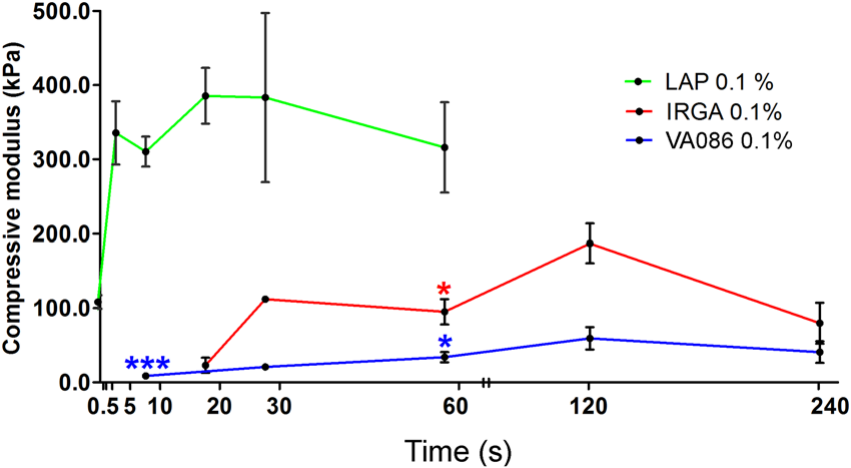
The LAP photo-initiator is able to generate crosslinked hydrogels with the highest modulus values at shortest exposure time (10 s). Compressive modulus (kPa) relative to mechanical properties of GelMa/HAMa, where crosslinking was obtained with three different PIs: Lithium-acylphosphinate (LAP); IRGACURE2959 (IRGA), and VA086 with different photocuring times. The moduli reached by LAP dependent crosslinking are several fold higher respect to the other PIs tested. IRGACURE2959 and VA086 do not achieve the moduli of LAP, even with longer exposure times. Error bars represent standard error of the mean between three replicates. The calculated statistical significance (p < 0.05) was obtained by unpaired *t* test.

Data obtained showed that the compression modulus of GelMa/HAMa hydrogels increase as a function of UV photocuring time (Fig. 1). However, the rates of reaction and the final modulus achieved dramatically differ between the three PIs. VA086 resulted in hydrogels with the lowest modulus, with its maximum of 60 kPa achieved at 120 seconds of light exposure. At shorter exposure times (10 s), the VA086 achieved a modulus of only 9 kPa while even shorter exposures did not result in stable crosslinking (i.e. the hydrogel remained in a liquid state). These poor mechanical properties are most likely due to the evolution of nitrogen species creating bubbling within the hydrogel^17^. IRGA resulted in considerably stronger hydrogels, achieving a compressive modulus of 190 kPa at 120 s exposure. At short exposure time (10 s), IRGA did not result in stable crosslinking, most likely due to oxygen inhibition of the photo-initiation reaction^22^. LAP achieved by far the highest modulus values (380 kPa at 20 s exposure), even at much shorter crosslinking time. IRGA and VA086 photo-activation do not lead to hydrogel hardening with short exposure times (0.5, 1, 2 and 5 s), while longer exposure times never equal the performance of LAP, which start to reach a plateau after only 5 seconds. Based on these data, and pending toxicity evaluation, we selected LAP as the potential PI of choice for use with the co-axial Biopen system.

**Figure 2:**
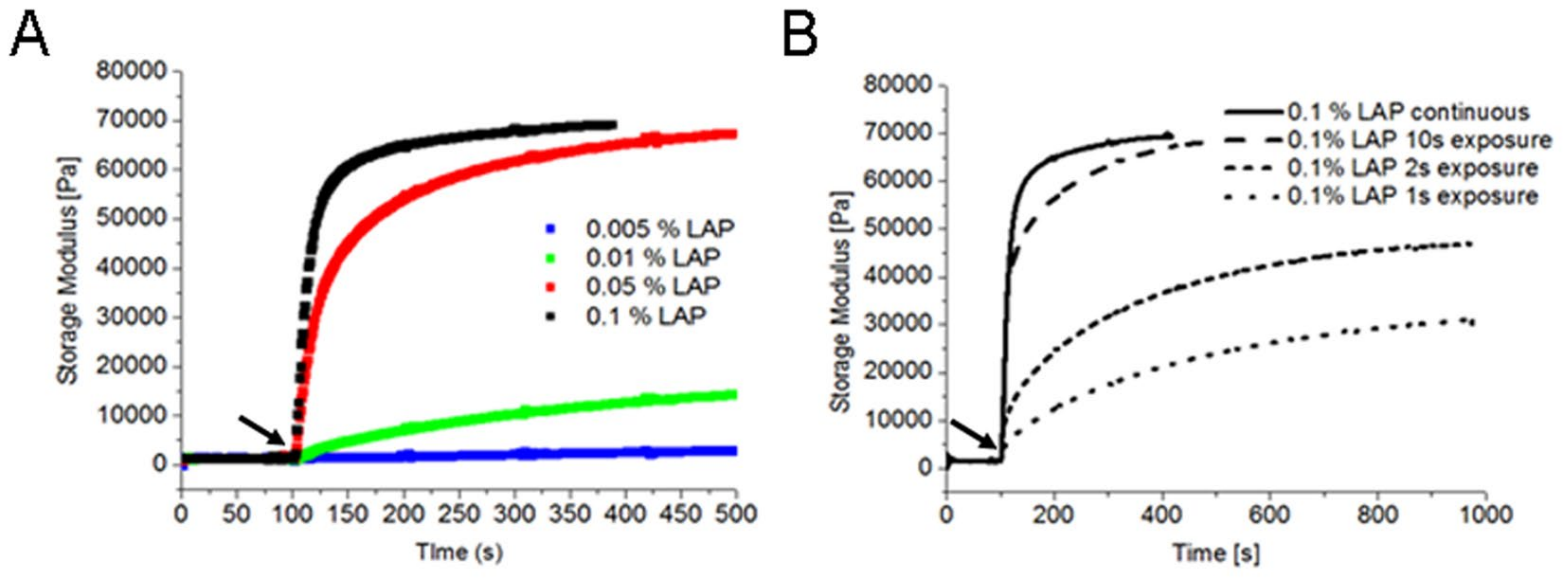
Higher concentration of LAP photoinitiator favour higher storage modulus. *In situ* photo-rheometry of GelMa/HAMa hydrogels incorporating LAP during the crosslinking reaction. Each plot shows storage modulus as a function of time with the UV light (365 nm, 100 mW/cm2) switched on at 100 s. (**A**) Storage modulus as a function of continuous UV exposure time for four concentrations of LAP (0.005% = open squares, 0.01% = light grey squares, 0.05% = grey squares and 0.1% = black squares). Both the reaction rate and the final storage modulus are impacted by LAP concentration. Low concentrations produce slow reactions resulting in low modulus hydrogels. The two highest LAP concentrations (0.05% and 0.1%) achieve comparable final modulus (~70 kPa), although the 0.05% concentration requires much longer exposure time to achieve this. (**B**) Effect of dark polymerization. GelMa/HAMa hydrogels incorporating 0.1% LAP were exposed to continuous UV exposure (thick black line) or single bursts of short times (10 s, 2 s and 1 s). The arrows indicate the point at which the UV light was switched off for the continuous exposure (100 s). During light exposure, all hydrogels follow similar crosslinking kinetics, however after the light turns off dark polymerization can continue for many minutes. In the case of the 10 s exposed hydrogel, the dark polymerization is sufficient to match the material crosslinked with a (much longer) continuous exposure.

*In situ* photo-rheology was then used to further characterize the rate of the photo-crosslinking reaction using the selected photo-initiator (LAP). This measurement records the storage modulus (which is proportional to the degree of crosslinking) as a function of time after the sample has been illuminated with 365 nm UV light. Figure 2A shows typical data obtained for four LAP concentrations (0.005%, 0.01%, 0.05% and 0.1%). All samples showed an increase in storage modulus as a function of time, though the rate of crosslinking increases with increasing LAP concentration. LAP 0.1% achieves close to its maximum storage modulus after approximately 60 s exposure, while LAP 0.05% requires more than 250 s to achieve maximum storage modulus. These data highlight the kinetic advantages of incorporating a higher concentration of PI (Note: these measurements were performed at an intensity of 100 mW/cm^2^). Figure 2B shows *in situ* photo-rheometry for LAP 0.1% at various exposure times. After 1 s of light exposure, the GelMa/HAMa material continues to crosslink via dark polymerization, achieving a storage modulus of 30 kPa at 1000 s exposure time. Meanwhile a light exposure of 10 s achieves a final storage modulus very close to that achieved for a similar sample exposed to continuous light exposure. Thus, the production of a 300kPa GelMa/HaMa hydrogel functionalized with 0.1% LAP can be achieved with a light exposure of only 10 s at 365 nm and 700 mW/cm^2^, thereby fulfilling the modulus requirement for *in situ* Biopen mediated deposition of co-axial cells-GelMa/HAMa during surgery.

#### Cell Viability: Photo-initiated crosslinking under long-wave UV (UV-A) irradiation

Having established LAP-mediated photo-initiation of GelMa/HAMa crosslinking as the method of choice for our intended application, we undertook cytotoxicity evaluation of the methodology, with particular focus on the photo-initiation process. The free-radical photo-crosslinking reaction involves three possible sources of cytotoxicity: (i) exposure to the PI, (ii) exposure to UV light, (iii) exposure to free radicals generated by UV-mediated photocuring of the PI. These were assessed for our system by 7 days’ growth of Adipose derived Stromal/Stem Cells (ADSCs) in the presence of: (i) LAP (0.1% w/v) on its own; (ii) UV exposure at 700 mW/cm^2^ and (iii) LAP with UV irradiation to activate the full photo-initiation process and its generation of free radical transients.

**Figure 3:**
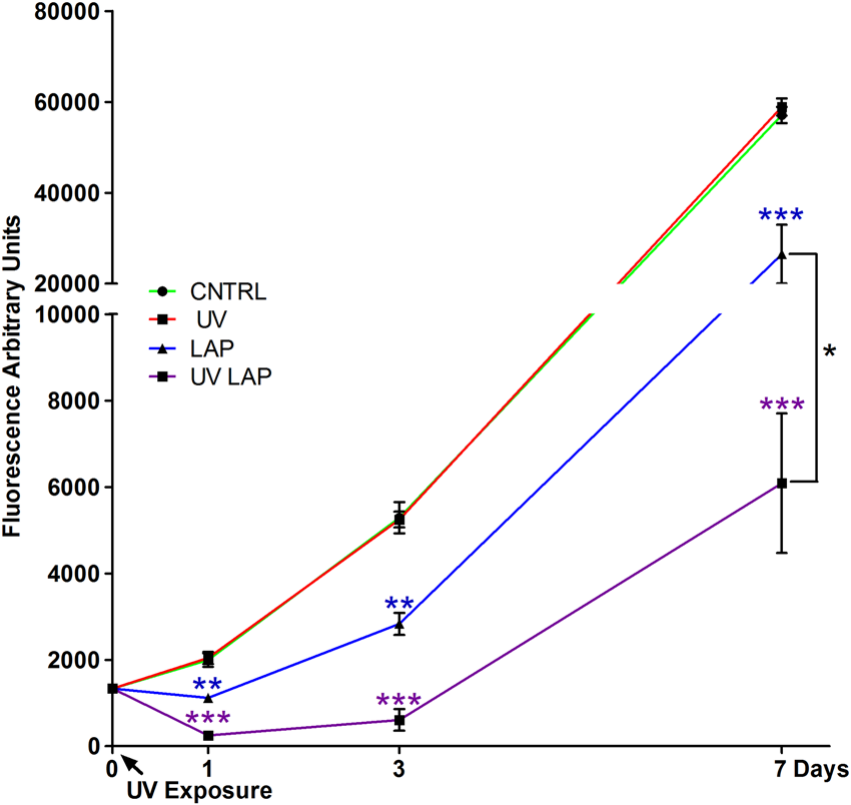
Cell cytotoxicity induced by PI and UV irradiated PI. ADSCs cultured in 2D and assayed along 7 days in culture with a metabolic test (Cell Titer-Blue®) to measure the cytotoxicity induced by cell exposure to UV light alone (UV), LAP on its own (LAP) and UV exposed LAP (LAP-UV) compared to untreated cells (CNTRL). Error bars represent standard error of the mean between three replicates. The calculated statistical significance was obtained by unpaired *t* test and calculated versus CNTRL. At day 7 statistics is calculated also for LAP-UV versus LAP.

UV light exposure at 700 mW/cm^2^ alone (without the presence of PI) did not significantly affect cell viability, with cell viability in irradiated cultures comparable to those of untreated (CNTRL) cells throughout the 7 days (Fig. 3). However, the possibility that in remaining viable, the cells had endured UV-irradiation damage and mutation cannot be excluded by our data. Cell populations exposed to LAP in absence of UV irradiation displayed a significant reduction in percentage viability (even at day 1), indicating that in itself, LAP was toxic to the cells. Further complexity of the LAP toxicity dynamic was evident upon cell exposure to LAP in the presence of UV light, which delivered significantly greater reduction in viable cell number compared to cells exposed to UV and LAP on their own even 1 day after exposure commenced. Exposure to UV light in presence of LAP, caused an eminent decrease in cell number respect to the control, which was not significatively different between all the concentrations tested (0.005%, 0.01%, 0.05% and 0.1%; Supp. Fig. 1A) so that the observed cytotoxicity was not reduced by exposing the cells to lower concentrations of LAP.

Despite evidence that the sub-population of cells left viable at day 1 post LAP/UV irradiation retained some proliferative ability, the live cell count was significantly lower than the LAP alone throughout the growth period, indicating that the initial LAP toxicity was exacerbated by photo-activation, most likely due to free radicals generated by LAP photo-activation-mediated degradation. This rationale gained further support by observation of eventual entry of cells exposed to photo-activated LAP to non-proliferative G_0_ cell cycle phase over the subsequent week post 7 days (day 15; Supp. Fig. 1B).

Under the same UV exposure conditions, the cell viability was also affected by the photoactivation of the two other PIs considered, IRGA and VA086 (Supp. Fig. 1B,C).

These data represent an “itemized” analysis of each component of our photo-polymerization system’s “in-process” potential to adversely affect ADSCs viability and thus provide a baseline “maximal” possible toxicity level. This is based on the premise that LAP diffusion (and therefore its bio-availability to the cells) will be significantly less in denser structures such as the cross-linked GelMa/HAMa hydrogels proposed for use in this work than in the liquid culture media used for this analysis. These data lay a comparative platform for evaluating if and, if so, how much of the toxicity of LAP photoactivation is eradicated by i) mono-axial and ii) co-axial encapsulation of ADSCs in GelMa/HAMa using our rapid Biopen-mediated process.

### The Core/Shell solution

Collectively, the photo-polymerization protocol defined above satisfy the modulus (at least to some degree) and viability criteria necessary for our application of ADSCs encapsulated in GelMa/HAMa hydrogel to repair osteochondral lesions *in situ* as part of our Biopen technology.

However, having established the significant (additive) toxicity of the LAP and photoactivation process on the ADSCs that we wished to use for chondrogenic differentiation, it remained to establish if and to what extent this PI toxicity translated to the full process.

**Figure 4:**
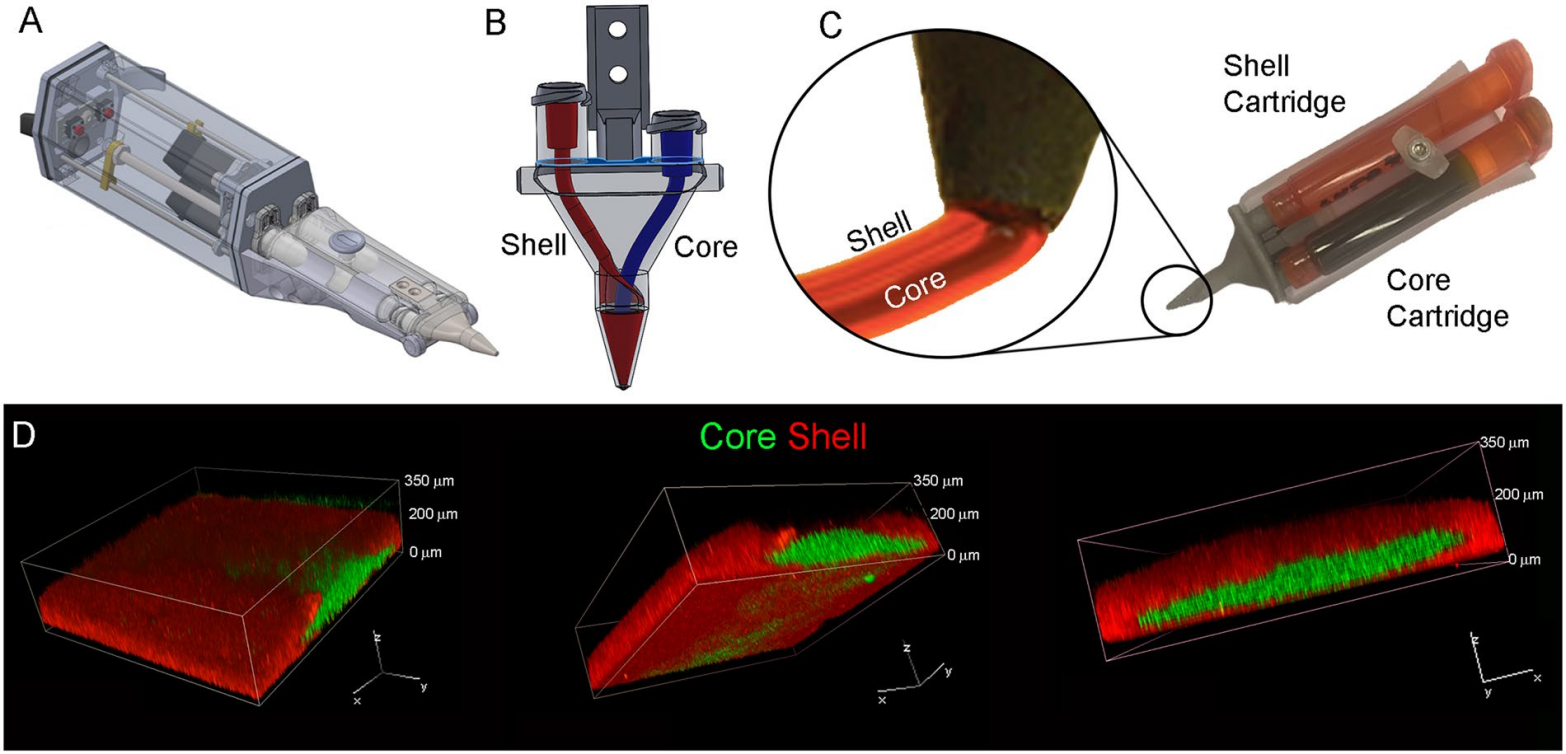
Core/Shell 3D printing by co-axial extrusion. (**A**) Schematic representation of the 3D co-axial handheld printer. (**B**) Schematic representation of the co-axial nozzle. (**C**) Picture of the cartridges dedicated to Core and Shell loading in the printer, with relative magnification of the nozzle during co-axial deposition. (**D**) Representative 3D rendered confocal images of Core/Shell printed sample labeled with fluorescent beads. The Shell (GelMa/HAMa plus LAP 0.1%) is shown in red channel, while the Core (GelMa/HAMa) is shown in green channel. *z*-stacks were acquired every 10 µm and 3D rendering was performed with NIS elements software using the *Alpha*-*blending* algorithm. A Nikon Plan Fluor 10x DIC L N1 NA0.3 objective lens was used. The panel shows the same image representative of 3D rendered of superimposed green and red channels in three different orientations.

Our approach postulated that the compound toxicity of LAP and photoactivation process should i) be considerably lessened by actual polymerization of the GelMa/HAMa in the first instance and ii) be further lessened by compartmentalization of the ADSCs in an inner, non-cross-linked GelMa/HAMa Core surrounded by a photo cross-linked Shell as part of a co-axial gelation process. Towards this end, we adapted our prototyped handheld Biopen printer (Fig. 4A)^20^ to eject an organized Core/Shell bioink in which the inner soft core, containing cells laden in non-cross-linked GelMa/HAMa, is surrounded by a robust shell made of cross-linked-GelMa/HAMa. This co-axial approach avoids the deposition of cells together with the photo-initiator, which will only be present in the shell. A dual concentric co-axial nozzle, characterized by an inner and outer orifice, enables the two different compartments (core and shell) to be dispensed (Fig. 4B,C), with a maximum resolution being 500 μm (Supp. Fig. 2A). On the basis of the mechanical and photo-rheology data obtained previously, we used a 0.1% w/v LAP concentration and crosslinking at 365 nm, 700 mW/cm^2^, at 10 s exposure time for shell hardening. In order to verify the Core/Shell printing capability of our device in the selected conditions, two GelMa/HAMa solutions were prepared with different coloured fluorescent beads before the 3D printing procedure. Confocal imaging analysis showed the core completely surrounded by the shell compartment with minimal apparent mixing of the two compartments (Fig. 4D) (the core is shown in green, the shell in red). Mechanical compression testing showed that the Core/Shell scaffold structure reached a Young’s Modulus of 195 KPa (±66.138). Thus, the generated Core/Shell scaffold presented “as-close-as-possible-to-optimal” mechanical characteristics for cartilage regeneration applications, giving rise to the possibility of building 3D cartilage-repair structures *in situ* during surgery (Supp. Fig. 2B,C).

### Cell behavior in the Core/Shell structure

**Figure 5:**
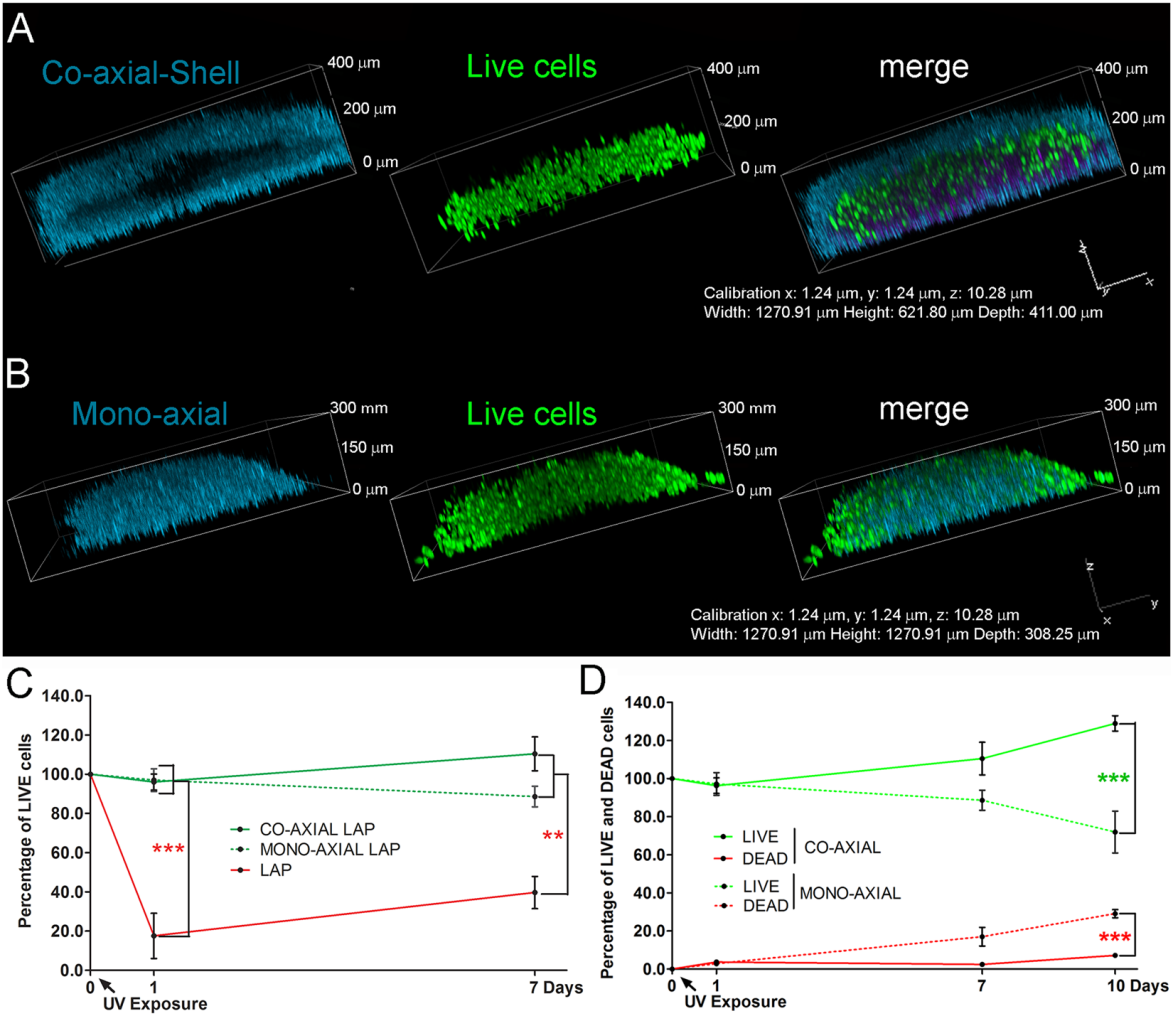
The co-axial printing produces 3D printed samples with high cell viability. (**A**,**B**) Representative 3D rendered confocal images of ADSCs Core/Shell (co-axial) and mono-axial bioprinted samples stained with Calcein-AM (live cells, green channel). The samples were labeled with fluorescent beads for identification of the Shell (GelMa/HAMa plus LAP 0.1%, cyan channel). Mono-axial samples were also labelled with fluorescent beads for analysis (GelMa/HAMa plus LAP 0.1% and ADSCs, cyan channel). *z*-stacks were acquired every 10 μm and 3D rendering was performed with NIS elements software using the *Alpha*-*blending* algorithm. Images show representative 3D rendered of single and superimposed (merge) cyan and green channels of confocal single 2D *z*-stacks. (**C**) The graph shows the quantification of Calcein-AM stained ADSCs to measure cytotoxicity over time in culture right after UV irradiation in co-axial (Core/Shell) and mono-axial 3D printed samples, and in 2D monolayer. The co-axial approach demonstrates higher cell viability and proliferation compared to mono-axial samples and 2D at 7 days post irradiation exposure. Error bars represent standard error of the mean between three replicates. Statistical significance (p < 0.05) was determined by One way Anova test with Dennett’s correction. (**D**) The graph shows the quantification of live and dead staining performed with Calcein-AM (live cells, green lines) and SYTOX (dead cells, red lines) to measure cytotoxicity over time in culture in both Core/Shell (co-axial, solid line) printed and mono-axial (dotted line) samples. The Core/Shell approach demonstrates significantly higher cell viability and proliferation compared to mono-axial samples at 10 days. Error bars represent standard error of the mean between three replicates. Statistical significance (p < 0.05) was determined by unpaired *t* test.

ADSCs were printed out in (i) mono-axial and (ii) co-axial photo-cross-linked GelMa/HAMa using the Biopen. The distribution and viability of cells within the respective structures were evaluated by Calcein-AM and SYTOX staining and fluorescence imaging (Fig. 5 and Supp. Fig. 3). Three-dimensionally rendered single *z* stacks obtained with confocal microscopy showed that after co-axial bioprinting, live and dead cells are mainly distributed in the Core (Fig. 5A and Supp. Fig. 3A,B). This confirmed our system’s ability to print a Core/Shell structure in which the ADSCs are compartmentalized to the Core, away from the LAP and photo-activation by-products. On the other hand, cell distribution in the mono-axial printed hydrogel showed both live and dead cells throughout the structure without any overt compartmentalisation (Fig. 5B and Supp. Fig. 3C,D).

Quantitative evaluation of live cells in these configurations over 7 days, demonstrated that the photopolymerization of GelMa/HAMa is able to shield the cytotoxic effect that stem from the direct contact with the photoinitiator and the generation of free radicals (Fig. 3C). Despite the shielding effect, a drop in viable cell numbers beyond the 7 days in the ADSCs was evident within the mono-axial construct (Fig. 3C,D). This suggests that the mono-axial photo-cross-linking configuration does not adequately protect the cells in the system from the toxicity generated by this printing process. In contrast to this, in the co-axial configuration the cells were observed to retain proliferative capability throughout the test period, indicating a protective effect imparted on the cells through the co-axial printing process (Fig. 3C). Further cell viability analysis along time in culture shows that compared to the mono-axial configuration the co-axial displays a steeper increase in cell number by day 10, reaching a 30% increase from the initial number (Fig. 3D). The mono-axial bioprinting configuration shows instead a viability decrease by 30% along with an increase in the number of dead cells (Fig. 3D).

Given the nature of the temporal cell viability dynamics observed within the co-axial structures, it is likely that this geometry results in a protective effect that emanates from removing the cells from exposure to the free radicals generated by the photo-activation process.

Taken together, our data clearly reflects the ability of co-axial Core/Shell bioprinting to better maintain the survival of stem cell niche.

**Figure 6:**
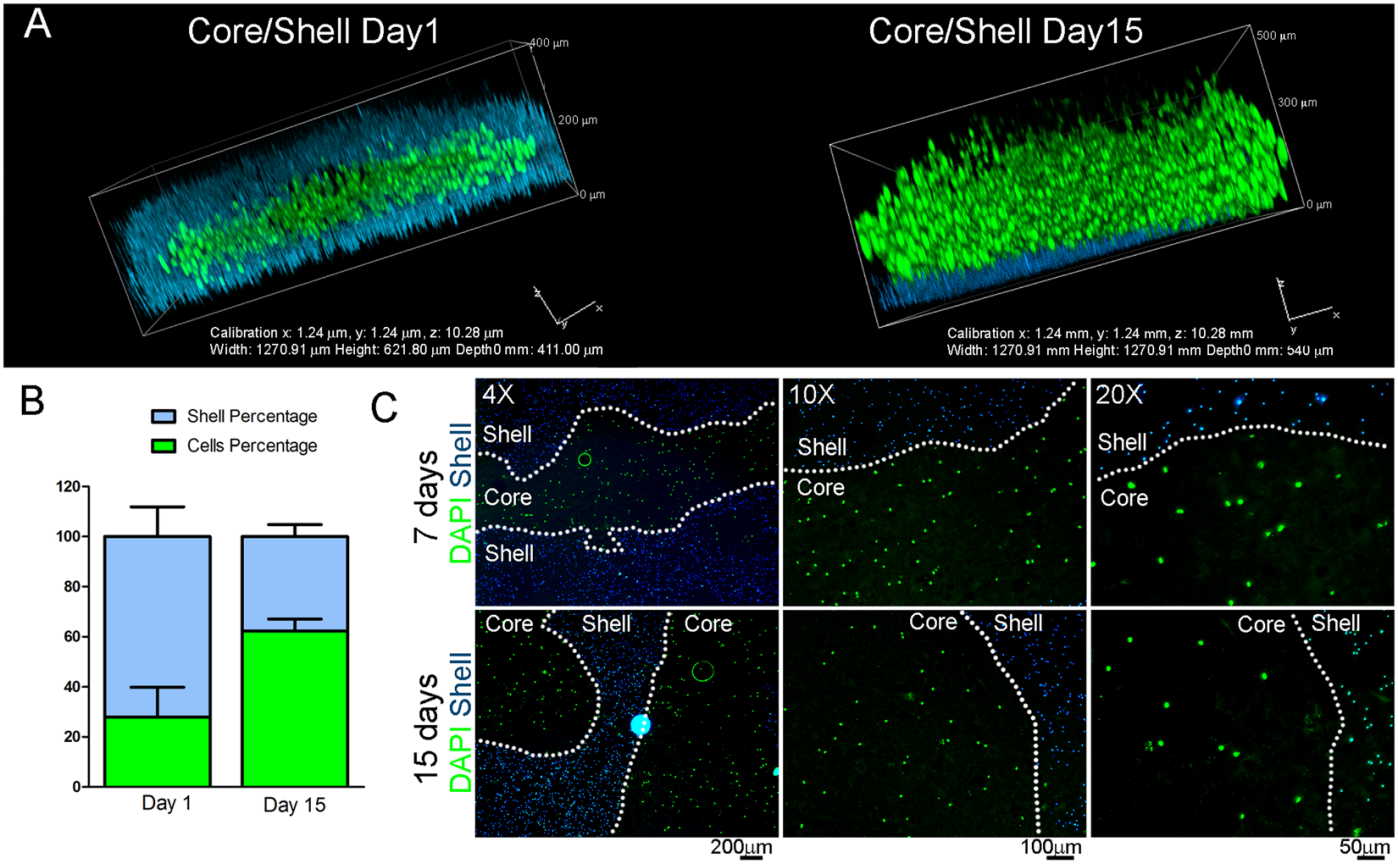
The Core/Shell design allows cells to proliferate. (**A**) Representative 3D rendered confocal images of ADSCs bioprinted samples stained with Calcein-AM (live cells, green channel) at day 1 and day 15 after printing. The Core/Shell samples were generated by labeling with fluorescent beads the Shell (GelMa/HAMa plus LAP 0.1%, cyan channel). *z*-stacks were acquired every 10 μm and 3D rendering was performed with NIS elements software using the *Alpha*-*blending* algorithm. A Nikon Plan Fluor 10x DIC L N1 NA0.3 objective lens was used. Images show representative 3D rendered of superimposed (merge) cyan and green channels of confocal single 2D *z*-stacks. (**B**) The graph shows the representation of the percentages of the same area occupied by cells (Cells percentage) and Shell (Shell percentage). (**C**) Fluorescence images of 10 μm cryosections obtained from the cryopreserved bioprinted samples. The cyan channel represents the labeled Shell, while cells have been stained with DAPI before imaging. A fluorescent Olympus IX70 inverted microscope with a SPOT Diagnostic RT-Slider camera and SPOT Diagnostic software was used. Olimpus 4X UPlanFL NA0.13, 10X CPlanFL RC NA0.3 and 20X LCPlanFL RC2 NA0.4 objective lenses were used.

**Figure 7:**
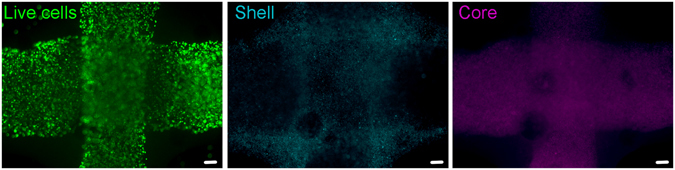
Core/Shell printing facilitates a layer by layer deposition of cell-laden hydrogels with structural integrity. Representative epifluorescent image of Calciein-AM (green channel) stained cells after 7 days from printing. The Shell and the Core has been differently labeled with fluorescent beads (cyan channel for Shell, violet channel for Core) to estimate the compartmentalization. An epifluorescent Olympus IX70 inverted microscope with a SPOT Diagnostic RT-Slider camera and SPOT Diagnostic software was used with an Olimpus 4X UPlanFL NA0.13 objective lens.

An additional feature of the Core/Shell bioprinting is that as time in culture progresses, the relative construct volume occupied by the cells considerably increase compared to the volume occupied by the shell (Fig. 6A, Supp. Fig. 4). Evident in Fig. 6B, the initial percentage of the space occupied by the shell decreases conversely to that occupied by the cells, providing good rationale for the expectation that likewise, if used surgically *in situ* to repair an osteochondral lesion, the co-axial structure would provide structural protection to the ADSCs within, allowing their expansion and chondrogenic differentiation. This lends support towards the further expectation that by eventually overcoming the physical constraint imposed by the GelMa/HAMa vehicle, the cells will be able to orientate into the structural layering required for the formation of functional hyaline cartilage. Fluorescence imaging analysis performed on single sections obtained from cryopreserved multilayered core/shell-printed samples, shows that the compartmentalization of the core is maintained for at least 15 days, since fluorescent beads are not internalized by cells despite the measured reduction of the shell (Fig. 6C). Thus, the increase in cell volume can be ascribed to the proliferation capability of the cells bioprinted within the Core. Layer by layer deposition using co-axial Core/Shell bioprinting allows building of composite 3D bioscaffolds if/when required. Epifluorescence imaging of layered “criss-cross” core/shell fibres shows live cells embedded in the core compartments along two distinctive layers, on the top of each other (Fig. 7, Live cells in green channel).

## Discussion

Our Biopen device was designed to print stem cells in 3D constructs directly into damaged cartilage during surgery *in situ*. To translate this freeform biofabrication tool into the clinical setting, in this work we aimed to define a bioprinting process that delivered a cell-laden structure with adequate structural integrity to support viable cell delivery to the highly compressive osteochondral lesion environment.

Assuring high rates of cell survival remains an important challenge in 3D bioprinting applications since it is critically related to the bioprinting process, the materials used and the intra- and post-printing chemical reactions/by-products. Hydrogel-based materials have been widely used as cell carriers and scaffolds in tissue engineering due to their structural compliance with natural extracellular matrix^23^. The extrusion bioprinting process necessitates a rapid phase transition from liquid during extrusion, to semi-solid to form a robust bioscaffold^24^. Matching the mechanical properties of the resulting bioscaffold to the target tissue is therefore important, since contained cellular behavior is, in part, and particularly for cartilage/chondrocytes, mediated by their mechanical microenvironment^25^.

Light-activated free-radical crosslinking (photo-polymerization) provides rapid reaction rates and generates uniform hydrogels with excellent temporal and spatial control of important properties such as mechanical stiffness. The degree of crosslinking, and hence mechanical strength of the printed scaffold, is a function of the reaction conditions (such as light intensity and exposure time) and the properties of the PI^22^. The most prevalent PI used in putative tissue engineering applications is 2-hydroxy-1-[4-(2- hydroxyethyl) phenyl]-2-methyl-1-propanone (IRGACURE^®^ 2959), owing to its water solubility and moderate cytotoxicity relative to other PIs^26^. However this PI was found to be toxic (in 2D culture, 30 minutes’ exposure to the PI in media and then between 3 and 7 minutes’ direct exposure to UV-A @ 4 mW/cm^2^) to different degrees in the various cell types evaluated and would require much longer UV exposure to photo-initiate cross-linking to the extent required for our proposed intra-surgical *in situ* application.

In recent years, other PIs have emerged as promising candidates for photo-crosslinking in the presence of cells. These include 2,20-Azobis[2-methyl-N-(2-hydroxyethyl) propionamide] (VA086), vitaminB2^27^ and lithium phenyl-2,4,6-trimethylbenzoylphosphinate (LAP). Whilst photo cross-linking with very high cell viability has indeed been achieved using VA086^28^, this again required very low-intensity exposure to 365 nm UV-A at 4 mW/cm^2^ (again, unacceptably long exposure time requirements for our purposes). Vitamin B2 has been proposed as a novel natural biocompatible photocrosslinking agents, nevertheless to obtain an ECM based material with 10/20kPa stiffness, a two-step photo/thermal process is required. The approach is thus not compatible with our settings that required a fast “real time” 3D bioprinting compatible with time for surgical procedures, and high stiffness for chondral based defect repair. Meanwhile, LAP has been advocated for its relative cytocompatibility and its ability to initiate crosslinking when exposed to higher (and therefore safer) wavelengths (>400 nm) of light^29^. Nevertheless, the photo cross-linking reaction remains the major potential source of cytotoxicity, and can therefore affect the final outcome of the designed application. Exposure to PIs, to UV light or to free radicals generated through light degradation of the PI, can all be adverse to cell viability (Fig. 3)^26^ and consequently, the (bio)functionality of the printed construct and the need for structural integrity (via degree of cross-linking) and cell viability need to be finely balanced in the adopted approach. Minimizing the PI concentrations and light intensity can alleviate cell toxicity but may reduce the efficiency of the crosslinking reaction, thus compromising the mechanical properties of the printed product. From this viewpoint, achieving adequate biomechanical properties thus requires longer crosslinking/UV exposure times (10–30 minutes), thereby limiting the clinical translatability of UV-cured polymers for *in situ* musculoskeletal tissue regeneration if associated in-process toxicity issues are not considered and accommodated.

Therefore given our intent to address the highly compressive osteochondral environment, the aim of our study was to develop a reliable method that allows rapid and efficient hydrogel hardening, while facilitating optimal cell survival. We reasoned that high cell viability and efficient hardening could be achieved by devoting different physical compartments to different structural components during the 3D printing process: one for cell deposition and one for structural integrity.

Consequently, we evaluated the crosslinking capacity of three PIs and selected 0.1% w/v LAP as the ideal PI for cross-linking GelMa/HAMa hydrogel to a modulus of ~300 kPa. This configuration allowed minimization of cell exposure to UV by application of a photocuring time of just 10 s with long-wave UV-A (365 nm) irradiation at 700 mW/cm^2^ UV (Figs 1 and 2).

Fairbanks and coauthors^29^ achieved high levels of post activation cell viability by crosslinking PEG-diacrylate using LAP with very low light intensity irradiation (10 mW/cm^2^), thereby reducing exposure to PI degradation-mediated free radical production. However, using the same conditions in our GelMa/HAMa hydrogel, would require a very long exposure time to reach a stiffness comparable to the 300kPa achieved in our system. Our approach sought an alternative means by which to preserve in-process cell viability without (excessively) compromising on the important cross-linkage-dependent stiffness requirement for *in situ* osteochondral repair application using our Biopen technology^20^.

We sought to achieve this protective effect by segregating the PI away from cells to maintain the most efficient photocuring conditions under a short time exposure (700 mW/cm^2^ for 10 s).

The selected photocuring settings used in this study allowed us to print cell-laden GelMa/HAMa hydrogel using our 3D Biopen device modified by incorporation of an extrusion nozzle designed for the co-axial deposition of materials (Fig. 4). This facilitated the geometric separation of two compartments of differing constitution in a Core/Shell configuration. By this process, we were able to extrude a soft non-cross-linked GelMa/HAMa core that contained ADSCs with an outer Shell component containing LAP PI that was photo cross-linked and provided structural support adequate for potential use within an osteochondral repair environment.

The co-axial cell/hydrogel structure that we generated successfully separated the ADSCs from the damaging effects of the LAP photo-activation of cross-linking. This was evident by high numbers (>95%) of viable cells in the co-axial structures immediately after the printing and photocuring steps and throughout all subsequent time in culture (Fig. 5). This was not observed in the mono-axial construct where the direct exposure of cells to the photo-activation process led to significant down-turn in cell viability. Furthermore, the co-axial process facilitated post-polymerization maintenance of cell proliferation within the structure, with progressive expansion of the relative volume occupied by the cells inside the overall structure (Fig. 6).

Various studies have reported co-axial 3D bioprinting^19, 30, 31^ to achieve structural integrity of the bioscaffold for deposition of hydrogels with low viscosity. Compared to those strategies, our approach is based on a new concept of co-axial Core/Shell geometry by which to protect printed cells from the fabrication process, thereby increasing the scope of materials that can be used for printing tissue constructs without compromising structural or cellular integrity. As such, our co-axial strategy geometrically compartmentalizes a solid phase that facilitates appropriate stiffness requirements and a viscous liquid phase that preserves cell viability by separating cells from PIs and the cytotoxic chemical by-products coming from the crosslinking reaction going on within the solid phase. In addition, the co-axial approach presented here uses a single hydrogel chemistry, which makes the system more readily translatable as a tool in the surgical field.

The results of the current study and of our previous work with this innovative Biopen printing device^20^ demonstrate that co-axial bioprinting has great potential for *in vivo* application directly at the surgical point of cartilage repair. The handheld 3D bioprinter’s ability to deposit co-axial cell-containing scaffolds lends favorably towards the potential for its eventual clinical translation, particularly in the field of musculoskeletal tissue regeneration and repair^32^. Specifically, with consideration of the nature of the GelMa/HAMa hydrogel and its mechanical properties, the Biopen-mediated bioprinting approach presented here has direct relevance to cartilage regeneration and repair, but will undoubtedly have applications in other areas when fully developed^12^. Further studies in this area will address the development and application of the co-axial Biopen approach described here to repair of cartilage in animal models as immediate precursor studies to clinical translation of this exciting technology.

## Methods

### Bioink preparation

Gelatin-methacryloyl/hyaluronic acid methacryloyl (GelMa/HAMa) was synthesized as previously described^20^. The materials was dissolved to a final concentration of 100 mg ml^−1^ GelMa and 20 mg ml^−1^ HAMa (10%GelMa-2%HAMa) in sterile PBS containing 100 U ml^−1^ penicillin and 100 μg ml^−1^ of streptomycin (GIBCO). Stem cells (ADSCs, see below for details of cells isolation and expansion) were mixed through the GelMa/HAMa to a final concentration of 5 × 10^6^ cells ml^−1^ and carefully loaded in the Core printing chamber. The Shell printing chamber was loaded with GelMa/HAMa and the indicated PIs.

### Photoinitiators and UV photocuring

The following three photoinitiators (PIs) were used to initiate photocrosslinking of the GelMa/HAMa hydrogel: VA086 (2, 2′-Azobis [2-methyl-N-(2 hydroxyethyl) propionamide]) from Sigma Aldrich; IRGACURE^®^ 2959 (IRGA2959) from Sigma Aldrich and lithium-acylphosphinate (LAP) from Tokyo Chemical Industry Co. (Tokyo, Japan).

Light irradiation was achieved using a 365 nm UV source (Omnicure LX400+, Lumen DynamixLDGI) fitted with a 12 mm lens (25 mm focal distance) at maximum intensity. The light source was placed directly on the bottom of the plastic wells where cells or bioprinted samples were deposited. Under these conditions, the light intensity was measured at 700 mW/cm^2^ through the plastic.

### Mechanical test

Flat discs (1 cm diameter, 1 mm thickness) of cured hydrogel were prepared by irradiating the hydrogel inside a Polydimethylsiloxane (PDMS) mold, covered with a thin glass coverslip. The tests were performed at room temperature using a TA Electroforce 5500 mechanical loading device (TA Instruments, New Castle, USA) fitted with a calibrated 5 lbf load cell. A 4.2 cm diameter compression plate was mounted on the mover. The protocol follows a procedure proposed by Loessner *et al*.^14^. The contact point with the bottom of a glass dish is recorded before the sample is placed in the same dish (in an unconfined environment) and compressed by a plate much larger than the nominal sample diameter (1 cm). Samples were hydrated by deposing droplets of PBS solution on their surface before testing. The displacement was controlled by a ramp function, lowering the compression plate at a rate of 0.01 mm/s, until a total displacement that is much larger than 15% of the sample height is achieved. Load, displacement and time are recorded from the test. The contact area between the sample with the compressing plate was measured using a Dino Lite 4111T microscope at 20X magnification and calibrated using a reference slide of known dimensions. Additionally, the point of inflexion of load versus time serves as contact point between the sample surface and the compression plate and gives the sample height. Subsequently, load and displacement are converted into stress (*σ*) and strain (*ε*) using the sample surface area and height. The compressive modulus was computed using stress data between 10 and 15% strain as follows: *E*_*c*_ = (*σ*_15_ − *σ*_10_)/(*ε*_15_ − *ε*_10_). This procedure was repeated for each of the 55 samples.

### *In situ* photo-rheology

All rheology experiments were performed on a Physica MCR 301 Rheometer (Anton Paar) in a parallel plate geometry (15 mm disk, 0.5 mm measuring distance) at room temperature (21–23 °C). For the flow experiments, the shear rate was ramped up from 0.1 to 100 s^−1^ over 5 minutes. A pre-shear of 5 s^−1^ for 2 minutes was introduced before the flow experiment to eliminate rheological history. Oscillatory measurements were performed at 1% strain and 1.5 Hz frequency. For *in situ* UV curing, light from the UV light source (Omnicure 1000, Lumen Dynamix LDGI) was routed through a 5mm optical fibre to illuminate the underside of the sample through a quartz crystal stage. The UV intensity (100 mW/cm^2^) was measured at the sample using a UV meter before and after each experiment. (Note: these rheology measurements were performed at an intensity of 100 mW/cm^2^ which was the maximum intensity achievable through the optical fibers on this system. The intensity used for other measurements in this study was generally 700 mW/cm^2^).

### Cell culture

Sheep Adipose Derived Stromal/Stem cells (ADSCs) were isolated from sheep infrapatellar fat pad (IPFP) as previously described^33^. After isolation, cells were maintained in culture media containing low glucose DMEM (St. Louis, LA, USA) supplemented with 10% FBS (GIBCO, Thermo Fisher Scientific Inc., Waltham, MA, USA), 100 U ml^−1^ Penicillin and 100 μg ml^−1^ Streptomycin solution (GIBCO), 2mM L-Glutamine (GIBCO), and 15 mM HEPES (GIBCO), 20 ng ml^−1^ epidermal growth factor (EGF) and 1 ng ml^−1^ fibroblast growth factor (FGF) (R&D Systems Inc., Minneapolis, MN, USA).

### Co-axial Core/Shell and unstructured mono-axial bioprinting

For co-axial Core/Shell 3D bioprinting, both chambers was loaded with GelMa/HAMa, but the LAP was added only to the Shell chamber diluted at 0.1% v/w. The extruded samples were then UV irradiated at room temperature, washed three times in PBS 1X and new complete culture medium was added in each well of a 24 were plates. To generate the unstructured mono-axial samples (no Core/Shell compartmentalization) the 3D extrusion was performed by using only 1 cartridge containing GelMa/HAMa, LAP 0.1% w/v and ADSCs. The samples were then UV irradiated at room temperature as described, washed three times in PBS 1X and new complete culture medium was added in each well.

### Cell viability assays in monolayer

In the 2D ADSCs cultures to determine the cytotoxicity, cells were plated at 2000 cell cm^−1^ in 48 well plates and let adhere O/N at 37 °C/5%CO2. Then for each well the three different PIs (LAP, VA086 and IRGA) were added at 0.1% in PBS v/w in a final volume of 0.5 mL/well, and for the LAP also at 0.005%, 0.01%, 0.05% and 0.1% v/w concentrations. After 10 minutes incubation at room temperature, the cells were UV irradiated as described in the previous paragraph, washed three times in PBS 1X and new complete culture medium was added in each well. To measure cell cytotoxicity during time in culture, the following cell viability tests were used: Calcein-AM staining (Thermo Fisher Scientific Inc.) for LAP, VA086 and IRGA comparison; CyQUANT® (Thermo Fisher Scientific Inc.) for the different concentration of LAP; Cell Titer-Blue® Cell viability assay (Promega, Madison, WI, USA) for the LAP by itself in comparison with UV light exposure and UV exposure alone. For CyQUANT and Cell Titer-Blue tests, cell viability was assessed using the manufacturer protocols in triplicate by acquiring fluorescent signal at each time point with a CLARIOSTAR microplate reader (BMG LABTECH, Ortenberg, Germany).

Live cell counts in cells monolayer experiments were performed in triplicate by acquiring epifluorescence images with Olympus IX70 inverted microscope with a SPOT camera and software using the indicated objective lenses.

### Core/Shell distribution and cell viability assays in mono and co-axial printed scaffolds

The imaging of Core/Shell fluorescent compartments were performed by incorporating fluorescent beads (Spherotech Inc., Lake Forest, IL, USA) into the Core (Blue beads) and Shell (Nile Red beads). Each bead type was 1.7–2.2 µm in diameter and was used at 0.1% w/v. Printed samples were then transferred onto 35 mm glass bottom dishes (MatTek Corporation, Ashland, MA, USA) for imaging. The mono-axial samples were printed containing Nile Red fluorescent beads only.

Cell viability in co-axial and mono-axial 3D bioprinting, was assessed using Calcein-AM and SYTOX^TM^ Green (Thermo Fisher Scientific Inc.), respectively to stain live and dead cells, according to the manufacturer protocols.

Confocal imaging was performed with NikonA1R confocal microscope using a Nikon Plan Fluor 10x DIC L N1 N.A. 0.3 objective lens. Digital images were processed using NIS-Elements software (Nikon, Amsterdam, Netherlands) without biased manipulations. 3D rendering was performed with NIS elements software using the *Alpha*-*blending* algorithm. All the images shown in this study are representative of at least three independent experiments.

Live and dead in the 3D a NikonTiE microscope equipped with a fully automated A1 confocal laser (A1R, Nikon, Amsterdam, Netherlands) and *NIS*-*Elements* software. The percentage of live and dead cells was calculated as follows: % of Live or Dead cells = 100 × n. Live or 100 n.Dead/TOT (n.Live + n.Dead) and 100% was normalized to day 0. An average of three different fields was counted per sample from at least three independent experiments.

Orthogonal projections obtained with the NIS-Elements software on the single *z*-stacks images were used for the representation of the volume occupied by Calcein-AM positive cells and fluorescent beads present in the Shell. The fluorescence measured from the two compartments at different time points was used to estimate their relative percentage over the same total area.

### Histological analysis

Samples were fixed in 1% paraformaldehyde (Santa Cruz Biotechnology, Dallas, TX, USA) for 4 hr at room temperature, embedded in O.C.T. TM Compound (Tissue-Tek, Sakura, Leiden, Netherlands) and flash frozen in liquid nitrogen. Cryosections of 10 μm thickness were mounted onto glass slides and stained with Safranin O (Sigma-Aldrich) for 10 minutes, dipped in 95% and 100% EtOH, cleared three times for 1 minute each in Xylene (Chem-Supply, GILLMAN, SA, Australia) and then mounted in Pertex medium (Grale HDS, Ringwood, VIC, Australia). For fluorescence analysis, 10 μm thickness slices were washed 2 times in PBS, permeabilized for 10 minutes in PBS-0.25%TritonX-100 (PBT) and then nuclei were stained by incubation with 5 µg/mL DAPI (Thermo Fisher Scientific Inc.) for 10 min at room temperature. The sections were washed in PBS, mounted with Fluoromount-G (Southern Biotech, Birmingham, AL, USA) onto glass slides and imaged using an epifluorescent Olympus IX70 inverted microscope using a SPOT Diagnostic RT-Slider camera and SPOT Diagnostic software using the indicated objective lenses. Images were processed using Photoshop software (Adobe).

### Statistical Analysis

All statistical analyses were performed using GraphPad Prism software^©^ with a statistical significance level of 0.05 indicated as p < 0.05. Differences between cytotoxicity of the PIs were determined using Unpaired *t* test or one-way Anova tests with Bonferroni or Dunnet corrections.

In all graphs stars represents * is p ≤ 0.05; ** is p ≤ 0.01; *** is p ≤ 0.001; not significant (n.s.) is p > 0.05.

### Ethical statement

Use of all animals and procedures (isolation of ADSCc from sheep infrapatellar fat pad) in this study was approved by the University of Melbourne Animal Ethics Committee [ID 1513586] and all the experiments were performed in accordance with relevant guidelines and regulations.

## Electronic supplementary material

Supplementary Information
