# Supplementary material for: Handheld Co-Axial Bioprinting: Application to in situ surgical cartilage repair
Source: Sci Rep. 2017 Jul 19;7:5837. doi: 10.1038/s41598-017-05699-x (PMC5517463; doi:10.1038/s41598-017-05699-x)
Supplement: Supplementary file 1 — Supplementary Information [file 41598_2017_5699_MOESM1_ESM.pdf]

Handheld Co-Axial Bioprinting: Application to *in situ* surgical cartilage repair

Serena Duchi, Carmine Onofrillo, Cathal D. O'Connell, Romane Blanchard, Cheryl Augustine, Anita F. Quigley, Robert M.I. Kapsa, Peter Pivonka, Gordon Wallace, Claudia Di Bella, Peter F.M. Choong

Supplementary Figures and legends

Supplementary Figure 1. Cytotoxicity induced by PIs and their UV activation.

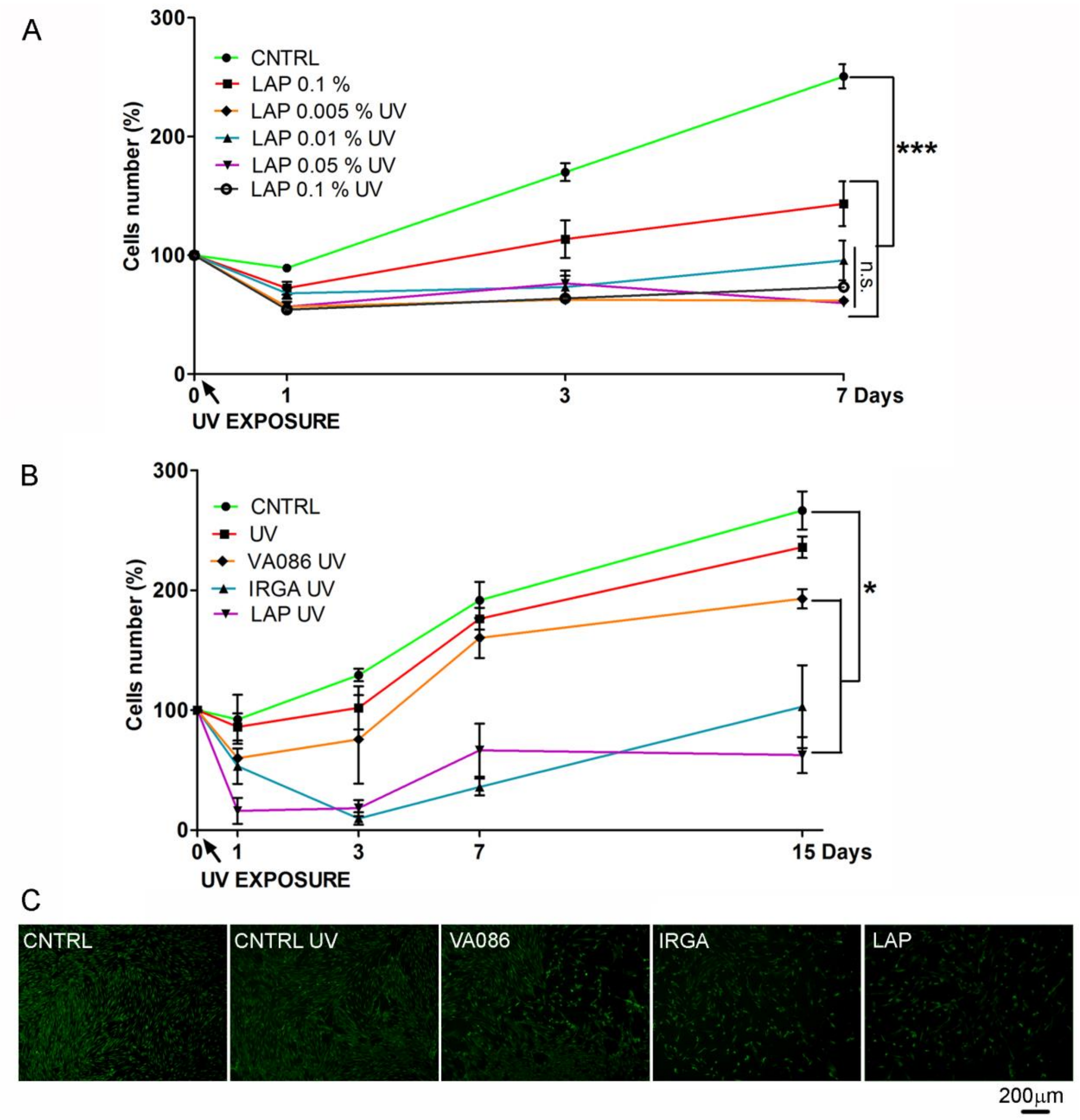

(A) ADSCs cultured in 2D and assayed with CyQUANT® to estimate the amount of cells after UV irradiation of different concentration of LAP until day 7 post photoactivation**.** The photocuring of LAP led to a cell number reduction that is not related to the concentration of the PI. LAP exposed samples without UV light (LAP 0.1%) affected cell viability only respect to the untreated control (CNTRL). Error bars represent standard error of the mean between three replicates. The calculated statistical significance ($p<0.05$) was obtained by one-way Anova with Bonferroni correction (different concentration of photocured LAP) or Dunnett's Correction (CNTRL versus all the other sample). **(B)** ADSCs cultured in 2D and assayed for cell viability with Calcein-AM to assess the cytotoxicity induced by the UV irradiation of three different PIs: Lithium-acylphosphinate (LAP); IRGACURE® 2959 (IRGA), and VA086 until day 15 post photoactivation. The three PIs show different degrees of toxicity post-light irradiation compared to the control (CNTRL). Exposure to UV light without the presence of PIs do not affected significatively cell viability (UV). Error bars represent standard error of the mean between three replicates. The calculated statistical significance ($p<0.05$) was obtained by one-way Anova with Dunnet's correction (CNTRL versus PIs). **(C)** Representative images of Calcein-AM staining of ADSCs cultured for 15 days after UV irradiation in presence of the three different PIs tested and under UV light on its own.

Supplementary Figure 2. Resolution and 3D printing capability.

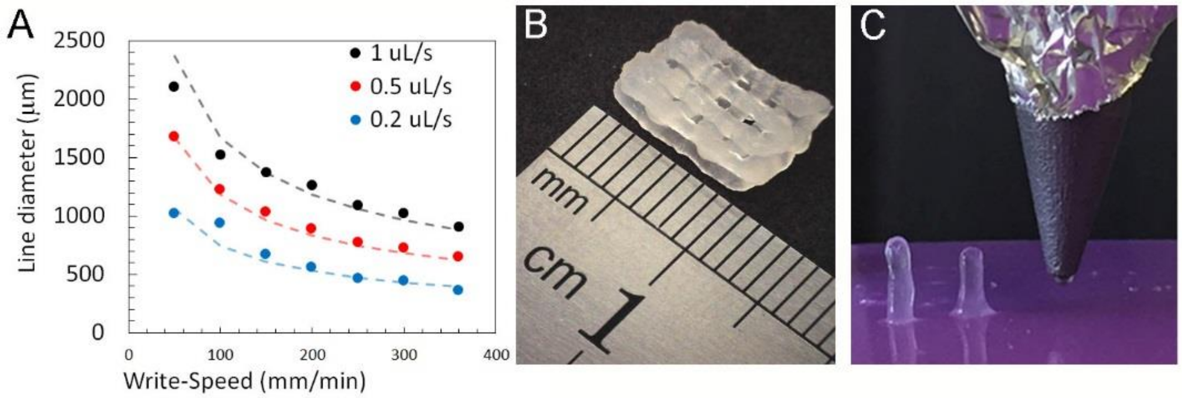

(A) The resolution of lines printed with GelMa/HAMa is controlled using the extrusion rate and the write-speed. The diameters of core-printed lines conform to that expected from a geometrically derived model (dashed lines). At low extrusion rates and high write speeds, minimum line diameters of less than 500 μm can be achieved. Self-supporting 3D structures **(B)** or pillars **(C)** can be prepared using Core/Shell extruded GelMa/HaMa hydrogel functionalized with 0.1% LAP with a light exposure of only 10 seconds at 365 nm and 700 mW/cm^2 right after single layer deposition.

Supplementary Figure 3. The co-axial configuration produces 3D printed bioscaffolds with higher cell viability compared to mono-axial configuration.

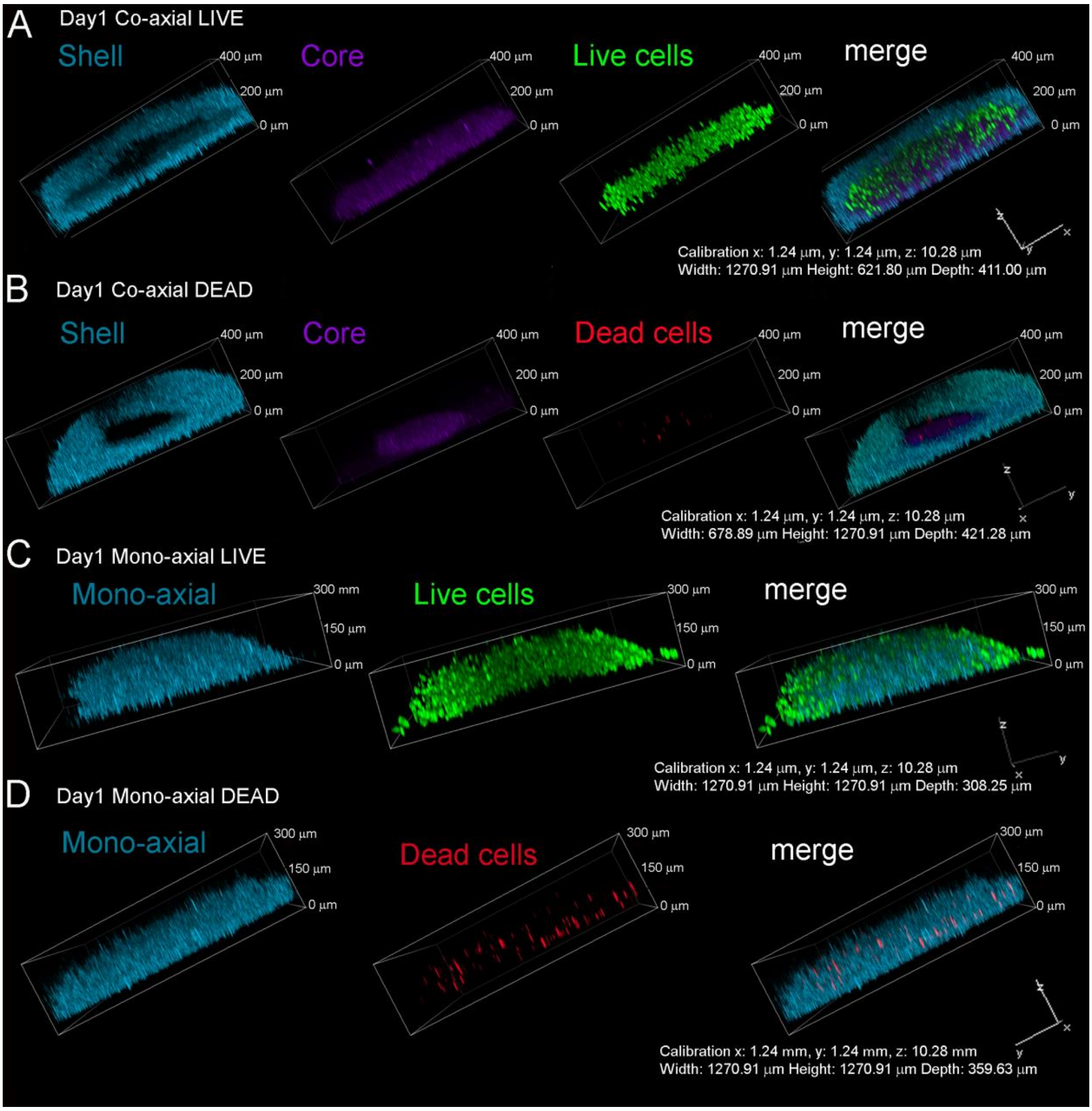

Representative 3D rendered confocal images of ADSCs Core/Shell bioprinted samples stained with (**A, C**) Calcein-AM (live cells, green channel) and (**B, D**) SYTOX (dead cells, red channel). The samples were labeled with fluorescent beads for identification of the Shell (GelMa/HAMa plus LAP 0.1%, cyan channel) and the Core (GelMa/HAMa plus ADSCs, violet channel). Unstructured samples were also labelled with fluorescent beads for analysis (GelMa/HAMa plus LAP 0.1% and ADSCs, cyan channel). *z*-stacks were acquired every 10 μm and 3D rendering was performed with NIS elements software using the *Alpha-blending* algorithm. Images show representative 3D rendered of single and superimposed (merge) cyan, violet and green/red channels of confocal single 2D *z*-stacks.

Supplementary Figure 4. The co-axial configuration allows cells to proliferate.

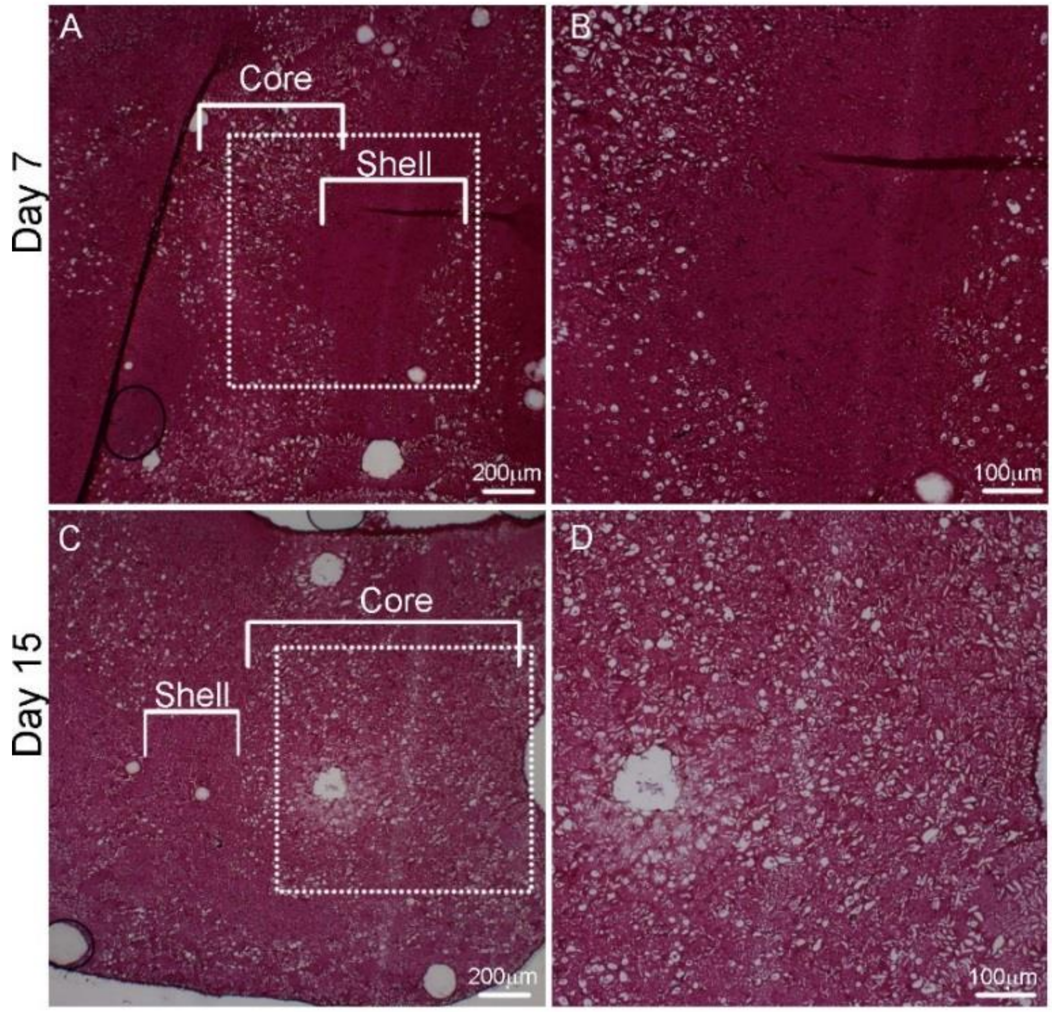

Representative images of histological analysis performed on Core/Shell (co-axial) bioprinted cryopreserved samples after day 7 (**A**, **B**) and day 15 (**C**, **D**) from printing. 10μm cryosectioned samples were stained with SafraninO. Despite the increase in the core volume, due to proliferation of cells, the compartmentalization is conserved during time in culture. An Olympus IX70 inverted microscope with a SPOT Diagnostic RT-Slider camera and SPOT Diagnostic software was used. Left panel images were captured with an Olympus 4X UPlanFL NA0.13 objective lens. (**C, D**) The images correspond to the areas specified by the dotted white square in panles **A** and **B**, which were captured with an Olympus 10X CPlanFL RC NA0.3 objective lens.
